# Calibration and test of contact parameters for alfalfa stalk at primary florescence based on discrete element method

**DOI:** 10.1371/journal.pone.0303064

**Published:** 2024-08-29

**Authors:** Tao Chen, Shujuan Yi, Yifei Li, Guixiang Tao, Xin Mao

**Affiliations:** College of Engineering, Heilongjiang Bayi Agricultural University, Daqing, Heilongjiang, China; University of Brescia: Universita degli Studi di Brescia, ITALY

## Abstract

In view of the lack of accurate models for discrete element simulation in the current research and development process of forage harvesting and crushing machinery, the contact parameters were calibrated based on Hertz-Mindlin (no slip) contact model by EDEM simulation software with alfalfa stalk at primary florescence as the research object. Based on the angle of repose, the restitution coefficient, static friction coefficient, rolling friction coefficient of alfalfa stalks were determined through the Placket-Burman test, steepest ascent test and Box-Behnken test. The simulation test of the repose angle was carried out with the determined contact parameters. The results showed that the relative error between the simulated repose angle and the physical test repose angle was 0.48%, which indicated that the calibrated contact parameters could truly reflect the physical characteristics of alfalfa stalks at the primary florescence. It provided a reliable model and parameter calibration method for the discrete element simulation in the research and development process of forage machinery, and also provided a reference for the research and optimization design of forage harvesting, crushing and processing machinery.

## Introduction

Livestock farming is a significant component of agriculture and has a significant impact on the Chinese economy. The development of livestock farming is based on the production and supply of pasture, which influences the scope and rate of development for livestock farming. The area of pasture planting in China is close to 150 million mu, accounting for 8.2% of the cultivated land. Among them, alfalfa planting area reached 50 million mu. The grass products mainly include hay, silage, grass blocks and grains, grass meal, straw and leaf protein. The main varieties of grass products are alfalfa, oats and leymus, of which alfalfa accounts for more than 70%. Alfalfa is one of the oldest and most important cultivated herbage in China. It is widely distributed in northwest, North and northeast China, and it is also cultivated in Jianghuai River basin. It is characterized by high yield, good quality and strong adaptability, and is the most economical cultivated forage grass, dubbed "the king of forage grass". Alfalfa can be used in many ways, including green feeding, grazing, adjusting hay, grass meal or silage, which is suitable for all kinds of livestock. The total annual output of forage is 90 billion kilograms, and the total output value is 12 billion dollars [1, 2].

The important component and hardest area of modern agricultural equipment digital design is the contact effect between agricultural machinery and diverse agricultural materials, and its influence on agricultural machinery design [3–5]. As a computer numerical simulation method based on the discontinuity assumption, the discrete element method (DEM) can be used to simulate and analyze the interaction between agricultural materials and mechanical equipment. The research and development cycle were shorten, and provide a new method for the digital design of modern agricultural equipment. It is currently widely applied in agricultural engineering [6–9].

Researchers around are gradually expanding their study on material parameter calibration in an effort to reduce the discrepancy between the discrete element simulation test and the actual test. Nguyen et al. (2020) analyzed the deviation between the shape of soybean seeds and spherical particles through digital image technology, and calibrated their discrete element simulation parameters in the EDEM software. The results show that spherical particles can be used to simulate the shape of soybean seeds [10]. Mousaviraad et al. (2017) established the discrete element simulation model of mature corn grain, which provided a reference for the simulation of screw grain transportation system [11]. Jalal et al. (2021) established apple’s model by EDEM software, and calibrated the restitution coefficient required for discrete element simulation by combined test and simulation [12]. Song et al. (2022) took mulberry soil as the research object, calibrated the contact parameters of soil particles by combined test and simulation [13]; Zhang et al. (2022) calibrated the restitution coefficient of mung bean seeds, though Hertz-Mindlin with bonding simulation model by used method of free fall collision, slope sliding and slope rolling respectively [14]. A discrete element simulation model of mung bean seeds was established. Ding et al. (2023) established the discrete element model of camellia oleifera seeds in the EDEM software by reverse engineering technology. The significant factors were screened through Placket-Burman Design and the steepest climbing test; The stacking angle was taken as the response value, the response surface (RSM) and machine learning were used to optimize the significance parameters. The research results showed that the camellia oleifera seed model and parameter calibration results established by the research could be used for discrete element simulation [15]. Existing research is mostly focused on crop stalks, soil, seeds, and other components, there is little research being done to build DEM models for forage stalks.

Alfalfa that commonly known as the "king of pasture" is a type of high-quality forage that has a wide range of uses and a planting area that accounts for nearly one-third of the total area of pasture. Squaring stage, primary florescence, and peak flowering stage are the three stages of alfalfa’s harvest. The peak flowering stage has the greatest yield, but the nutritional value is very low. Although the nutritional value is highest in the squaring stage, the yield is minimal, but the yield is low in this stage. Therefore, most farmers choose to harvest alfalfa in the early flowering stage with good quality and high yield [16]. In this study, the primary florescence of alfalfa stalk is used as the research object. The discrete element model of the alfalfa stalk is built based on the Hertz-Mindlin (no slip) contact model by EDEM. Base on the Plackett-Burman test, steepest ascent test, and Box-Behnken test, the contact parameters of the alfalfa stalk are determined. Including the restitution coefficient, static friction coefficient, and rolling friction coefficient. It offers a general method for calibrating discrete element parameters for alfalfa and other pasture during various harvest periods and serves as a reference for the development of forage harvesting, crushing, and processing machinery as well as for institutions that deal with transportation and feeding.

## Materials and methods

### Test material

**Fig 1:**
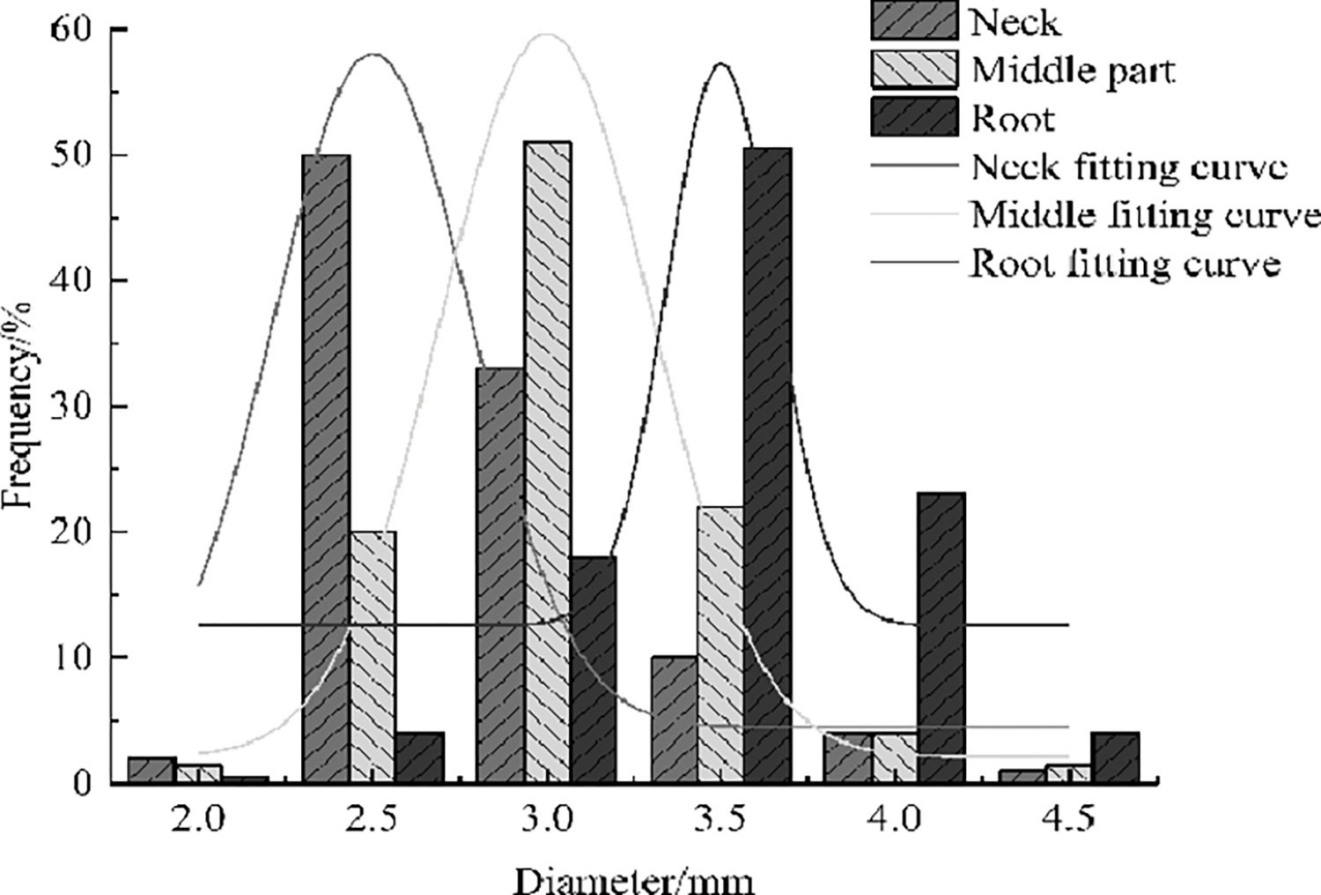
Stem diameter distribution of alfalfa in early flowering period.

The stalks of alfalfa used in the experiment were selected from the dry grass grass planting base of Duerbert County, Daqing City, Heilongjiang Province. The collected alfalfa had no pests and diseases, no obvious mechanical damage, and cut the branches and leaves in the natural state at the initial flowering stage. The average water content was 68.7%, and the diameter distribution of stems was shown in Fig 1.

The average diameter and length of alfalfa stem root were 3.48mm and 134.6mm respectively. The average diameter of the middle is 3.16mm, the length is 156.1mm; The average diameter of the neck is 2.92mm and the length is 163.3mm.

#### Determination of material parameters

**Fig 2:**
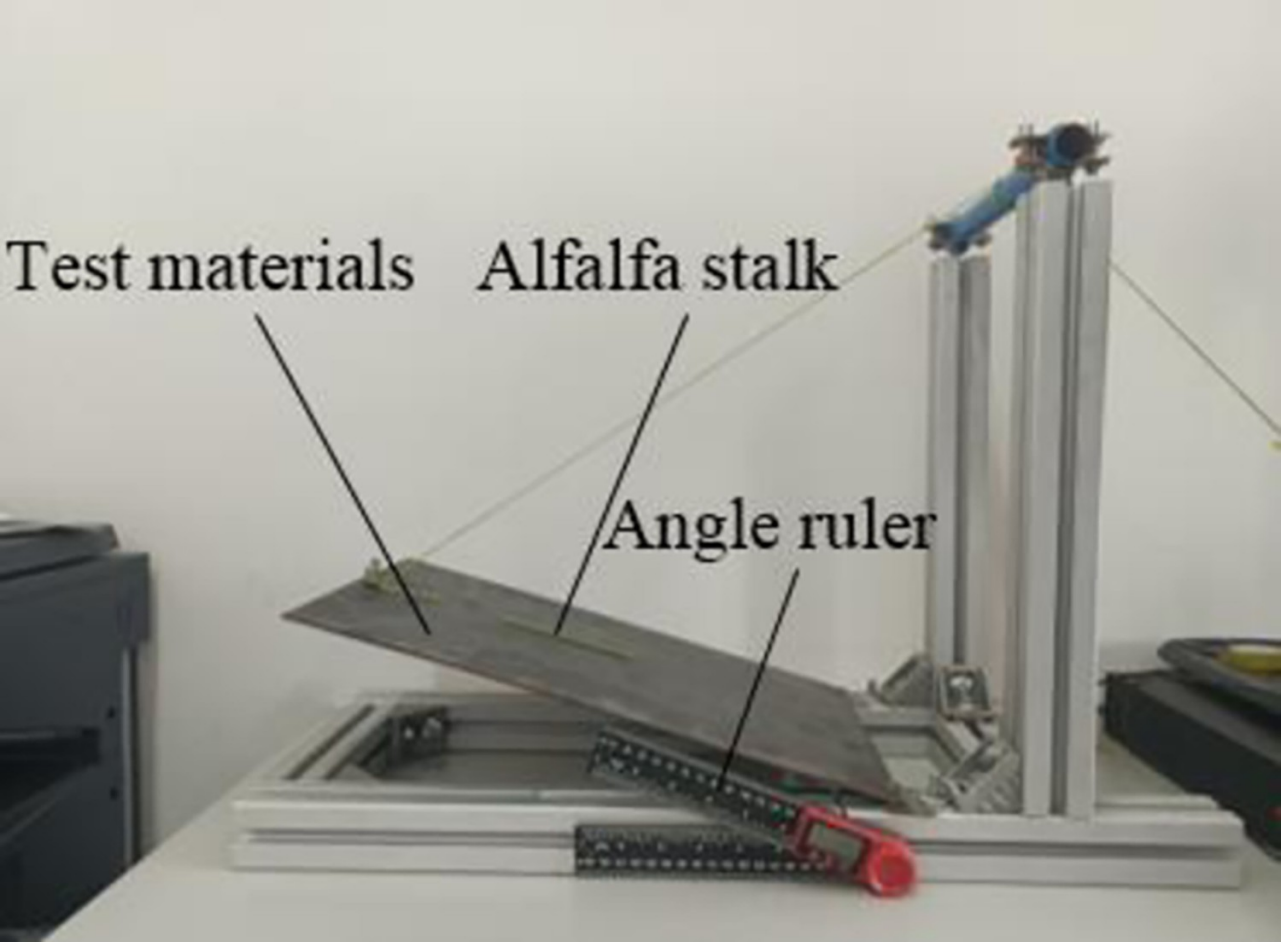
Measuring device for static friction coefficient of alfalfa stalk.

*Static friction coefficient*. In this study, a simple static friction coefficient test platform was built to obtain the static friction coefficient of alfalfa stalk-alfalfa stalk and alfalfa stalk-steel. The measuring device was shown in Fig 2. Before the test, the base of the inclinometer was placed horizontally and the measuring plane was adjusted to a horizontal state [17].


$$f_{s}=\tan\alpha$$ (1)

Where fs is static friction coefficient of alfalfa stalk; α is Critical angle of static friction coefficient (°).

When the static friction coefficient of alfalfa stalk-alfalfa stalk was measured, the steel was replaced with evenly arranged alfalfa stalks. By multiple measurements, the static friction coefficient of alfalfa stalk-alfalfa stalk was 0.4~0.6, and the static friction coefficient of alfalfa stalk-steel was 0.3~0.7.

**Fig 3:**
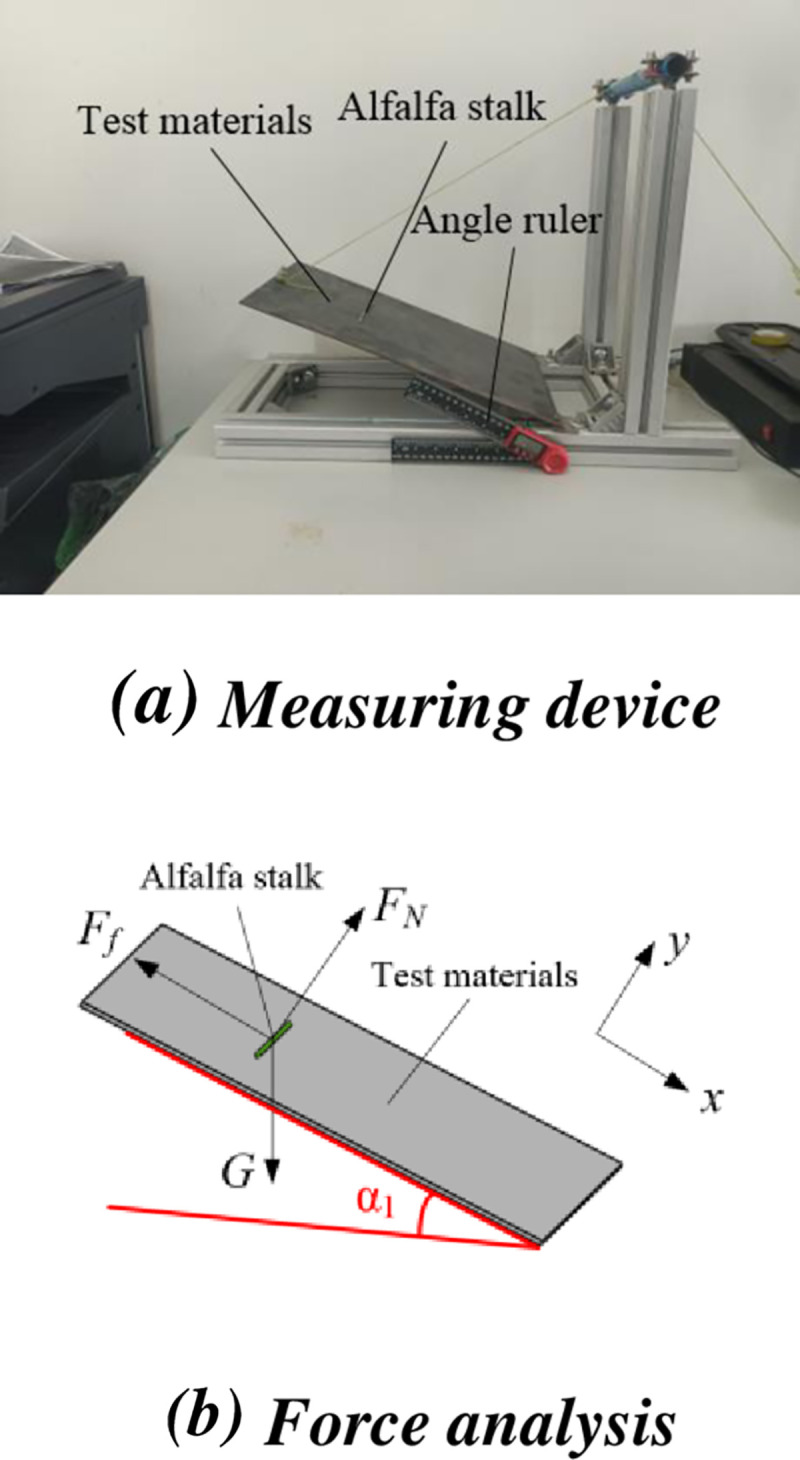
Alfalfa rolling friction coefficient measurement device and force analysis diagram. (a) Measuring device. (b) Force analysis.

*Rolling friction coefficient*. The alfalfa stalk was placed radially along the length direction of the measuring plane on the steel plate, the measuring plane was turned slowly clockwise and stopped rotating when the alfalfa stalk just rolled [18]. The inclination angle of the measuring plane was measured by protractor. The measuring device and force analysis were shown in Fig 3.

During the rolling process of alfalfa stalk, the rolling friction couple moment on the slope was proportional to the supporting force of the slope. When the slope is inclined to a certain degree, the alfalfa stalk will have a rolling trend. It could be obtained from force analysis.


$$M=f\cdot F_{N}$$ (2)

$$F_{N}-G\cos\alpha_{1}=0$$ (3)

$$Gr\sin\alpha_{1}-M=0$$ (4)

$$f=\frac{M}{F_{N}}=r\tan\alpha_{1}$$ (5)

Where M is rolling friction couple moment (N·m); f is rolling friction coefficient; F_N_ is supporting force of alfalfa stalk on an oblique plane (N); G is gravity of alfalfa stalk (N); α_1_ is critical angle of rolling friction of alfalfa stalk (°); R is radius of alfalfa stalk (mm).

When the rolling friction coefficient between alfalfa stalk-alfalfa stalk was measured, the steel plate could be replaced by evenly arranged rows of alfalfa stalks. Through many tests, it cloud be obtained that the rolling friction coefficient of alfalfa stalk-alfalfa stalk was between 0.05~0.25, and the rolling friction coefficient of alfalfa stalk-steel was between 0.1~0.3.

**Fig 4:**
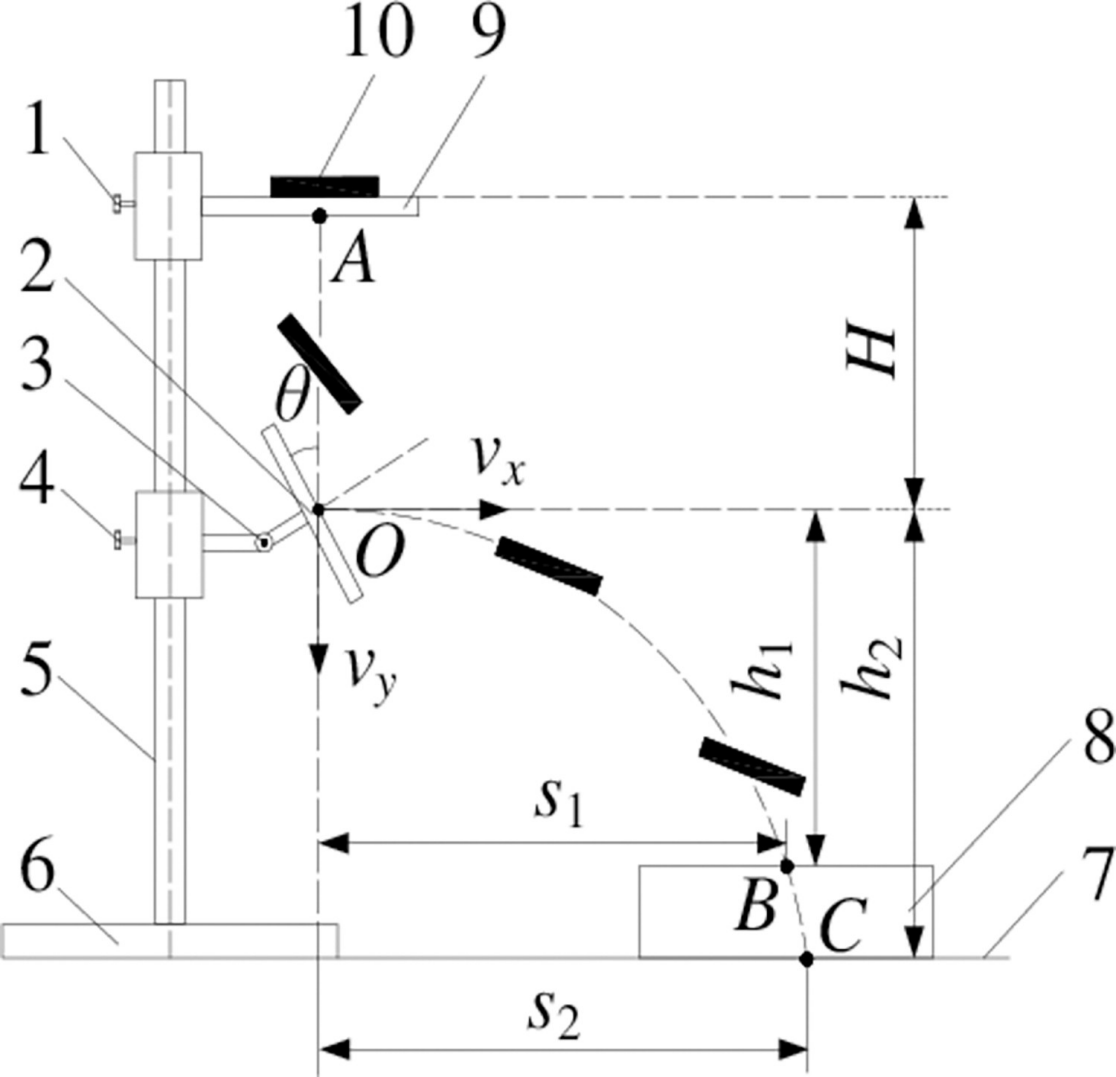
Measurement device and motion analysis of alfalfa stalk restitution coefficient.

*Restitution coefficient*. The restitution coefficient is a parameter to measure the ability of the object to recover its original shape after denaturation. It was the ratio of the instantaneous normal separation velocity of the contact point at the end of the collision to the normal approaching velocity before the collision [19]. The measuring principle was shown in Fig 4.

During the test, the alfalfa stalk fell from the blanking hole with the height of H of the impact plate and be free fall, colliding with the impact plate below. The inclination angle of the impact plate can be adjusted, and the impact material on the plate can be changed depending on the test’s purpose. The alfalfa stalk was only effected by gravity in this process if air resistance was disregarded. According to the kinematics principle, the formula for calculating the instantaneous velocity of alfalfa stalk before contact and collision with the impact plate is shown in Eq 6.


$$\left\{ \begin{array}{l} v_{0}=gt\\ H=\frac{1}{2}gt^{2} \end{array}\right.$$ (6)

Where v_0_ is speed of alfalfa stalk before collision (m/s); t is free fall time of alfalfa stalk (s).

It can be known from Formula (6) that the instantaneous speed v_0_ of alfalfa stalk before impact at the impact plate point O was:

$$v_{0}=\sqrt{2gH}$$ (7)

With the geometric shape of the alfalfa stalk was ignored and assumed that the trajectory of the alfalfa stalk rebound was a horizontal throwing motion. A uniform linear motion with a component speed of v_x_ in the horizontal direction, and a uniform accelerated linear motion with a component speed of v_y_ in the vertical direction, it was possible to conclude that the trajectory of the alfalfa stalk rebound was a parabola. According to the principle of horizontal throwing motion:

$$\left\{ \begin{array}{l} s=v_{x}t_{1}\\ h=v_{y}t+\frac{1}{2}gt_{1}^{2} \end{array}\right.$$ (8)

Where t_1_ is alfalfa stalk rebound movement time (s); s is displacement of alfalfa stalk in horizontal direction (m); h is free fall height of alfalfa stalk after collision (m).

It was difficult to accurately obtain the horizontal and vertical partial velocities v_x_ and v_y_ after rebounded, since the time t from the free fall of alfalfa stalks at the blanking port to the collision with the impact plate was measured difficultly. The height of the receiving plate was adjusted, and the test was carried out for two times. Taking pictures of the rebound trajectory of alfalfa stalks under two groups of different receiving tray heights with high-speed cameras. The horizontal displacement s_1_, s_2_ and vertical displacement h_1_, h_2_ were measured respectively. The equation for calculating the horizontal and vertical partial speeds v_x_ and v_y_ of the alfalfa stalk was:

$$\left\{ \begin{array}{l} v_{x}=\sqrt{\frac{gs_{1}s_{2}(s_{1}-s_{2})}{2(h_{1}s_{2}-h_{2}s_{1})}}\\ v_{y}=\frac{h_{1}v_{x}}{s_{1}}-\frac{gs_{1}}{2v_{x}} \end{array}\right.$$ (9)

According to the definition of restitution coefficient, the calculation formula was:

$$e=\frac{v_{n}}{v_{0n}}=\frac{\sqrt{v_{x}^{2}+v_{y}^{2}}\cos(\theta +\arctan\frac{v_{y}}{v_{x}})}{v_{0}\sin\theta }$$ (10)

Where e is restitution coefficient; v_n_ is normal separation velocity after collision (m/s); v_0n_ is normal approach velocity before collision (m/s);θ is inclination angle of impact plate (°).

After many experiments and measurements, the restitution coefficient of alfalfa stalk-alfalfa stalk was found to be between 0.35~0.55, and the restitution coefficient of alfalfa stalk-steel was between 0.3~0.5.

**Fig 5:**
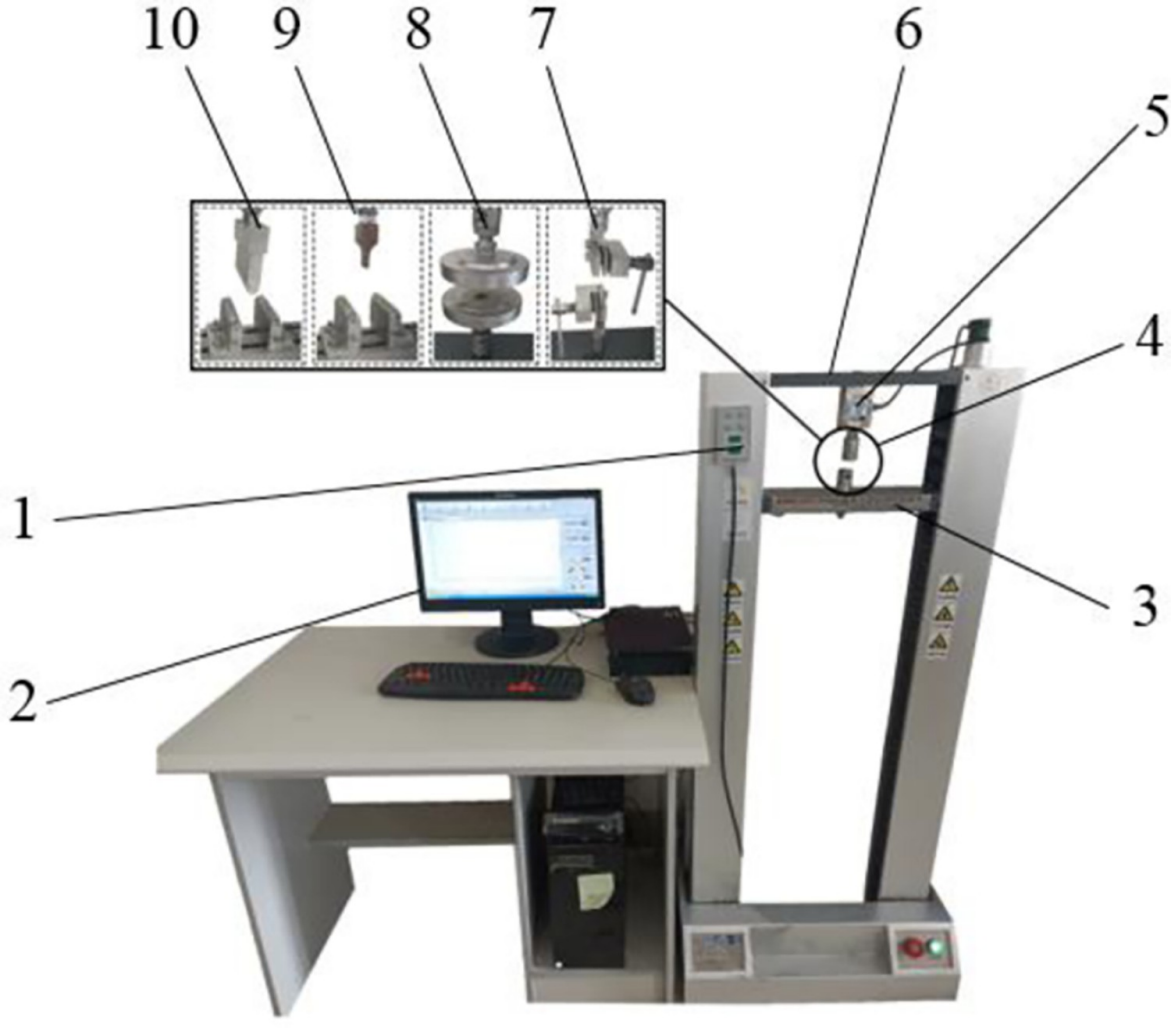
CTM2050 microcomputer-controlled electronic mechanics universal testing machine.

*Poisson’s ratio and shear modulus*. In this paper, the CTM2050 universal mechanical testing machine is used to measure the Poisson’s ratio and shear modulus of alfalfa stalks. The test apparatus is shown in Fig 5.

Poisson’s ratio refers to the ratio of the absolute value of the radial normal strain to the axial normal strain of the material under uniaxial tension or compression, also known as the transverse deformation coefficient, which is the elastic constant reflecting the transverse deformation of the material [20, 21]. The definition method is used to measure the Poisson’s ratio of alfalfa stalks, and the calculation formula is

$$\mu =|\frac{\varepsilon_{x}}{\varepsilon_{y}}|=\frac{\Delta L/L}{\Delta H/H}$$ (11)

Where μ is Poisson’s ratio; ε_x_ is radial strain of alfalfa stalk; ε_y_ is axial strain of alfalfa stalk; ΔL is absolute radial deformation of the alfalfa stalk (mm); L is initial diameter of alfalfa stalk (mm); ΔH is absolute axial deformation of alfalfa stalk (mm); H is initial length of the alfalfa stalk (mm).

**Fig 6:**
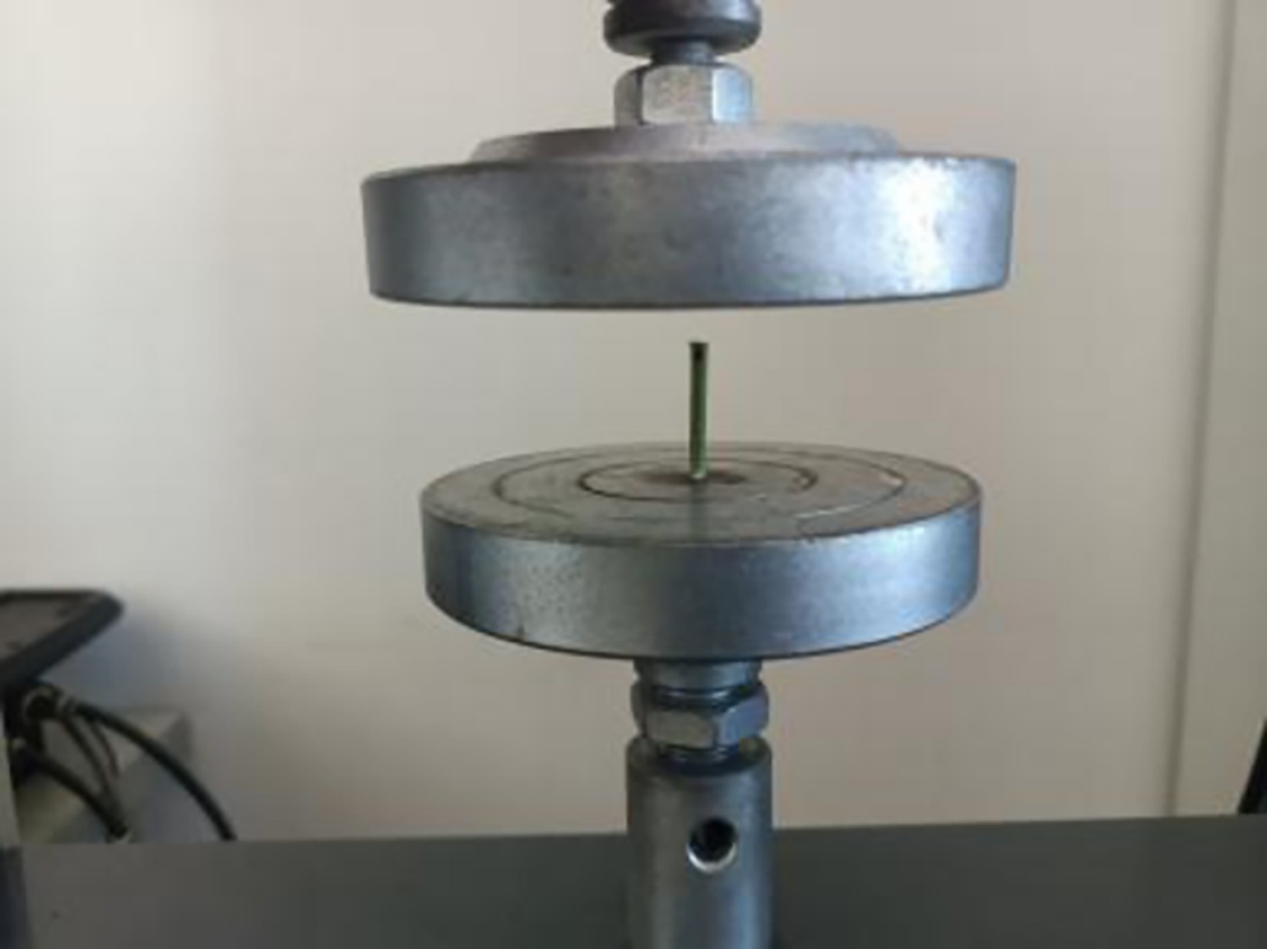
Alfalfa stalk compression test.

The alfalfa stalk with a length of 10mm and an average diameter of 3.3mm was made as the test material. During the compression test, the loading speed was set at 0.5mm/s, and the loading time was 10s. After the loading, the radial and axial deformation of the alfalfa stalk was measured with a digital vernier caliper, and the test was repeated for five times. The compression process is shown in Fig 6.

The Poisson’s ratio of alfalfa stems calculated from Eq (11) is 0.38.

Shear modulus is the ratio of shear stress to shear strain of a material under the action of shear stress within the limit of elastic deformation ratio. It represents the ability of the material to resist shear strain [22, 23], and the calculation formula is shown in Eq 12.


$$\left\{ \begin{array}{l} G_{1}=\frac{E}{2(1+\mu )}\\ E=\frac{\sigma }{\varepsilon } \end{array}\right.$$ (12)

Where G_1_ is shear modulus of alfalfa stalk (Pa); E is elastic modulus of alfalfa stalk (Pa); σ is maximum compressive stress (Pa); ε is linear strain.

According to the compression test, the elastic modulus of alfalfa stalk is about 43.5 MPa and the shear modulus is about 15.8 MPa calculated by Formula (12).

#### Repose angle model

**Fig 7:**
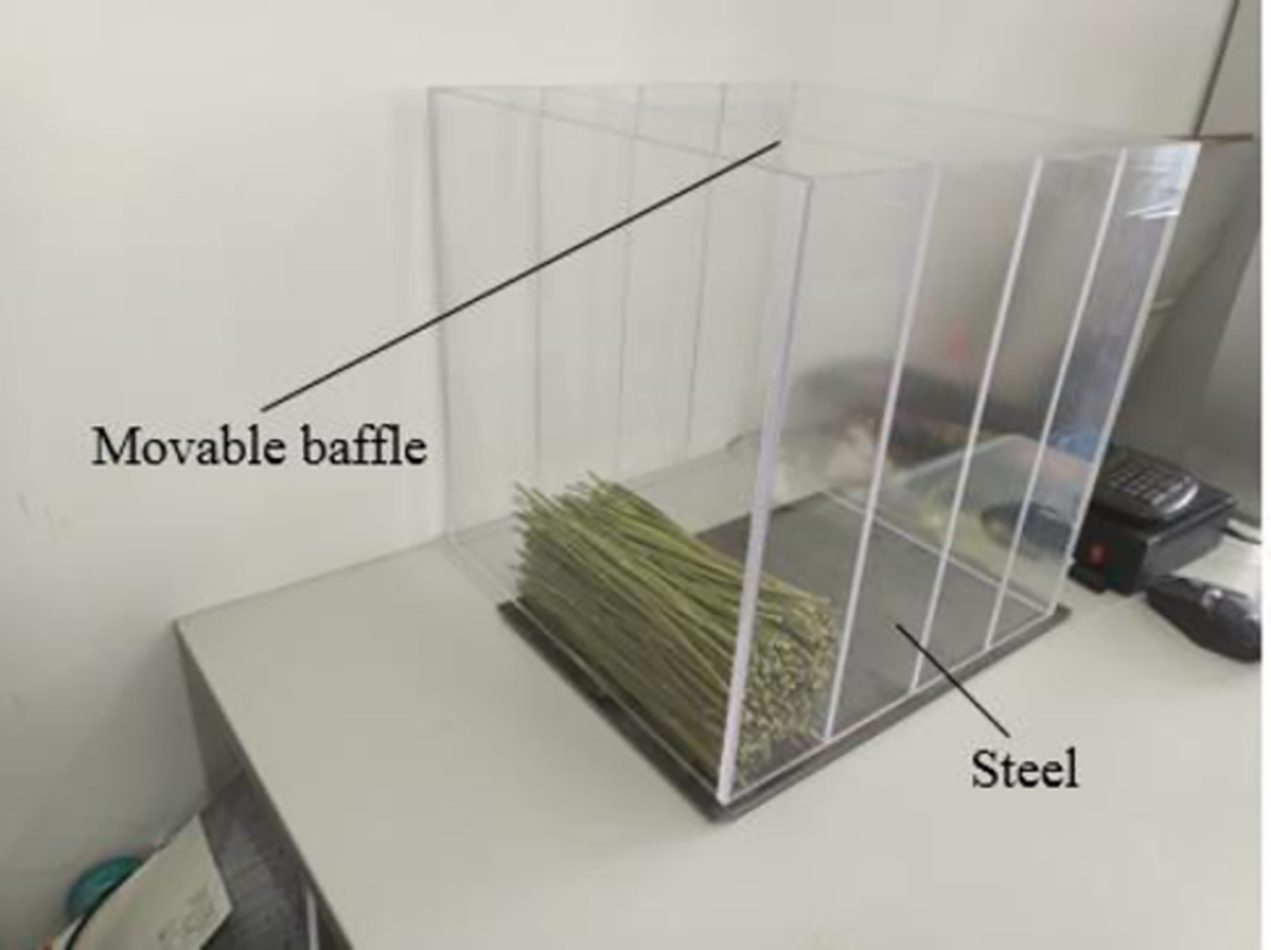
Experiment of alfalfa stalk angle of repose measurement.

**Fig 8:**
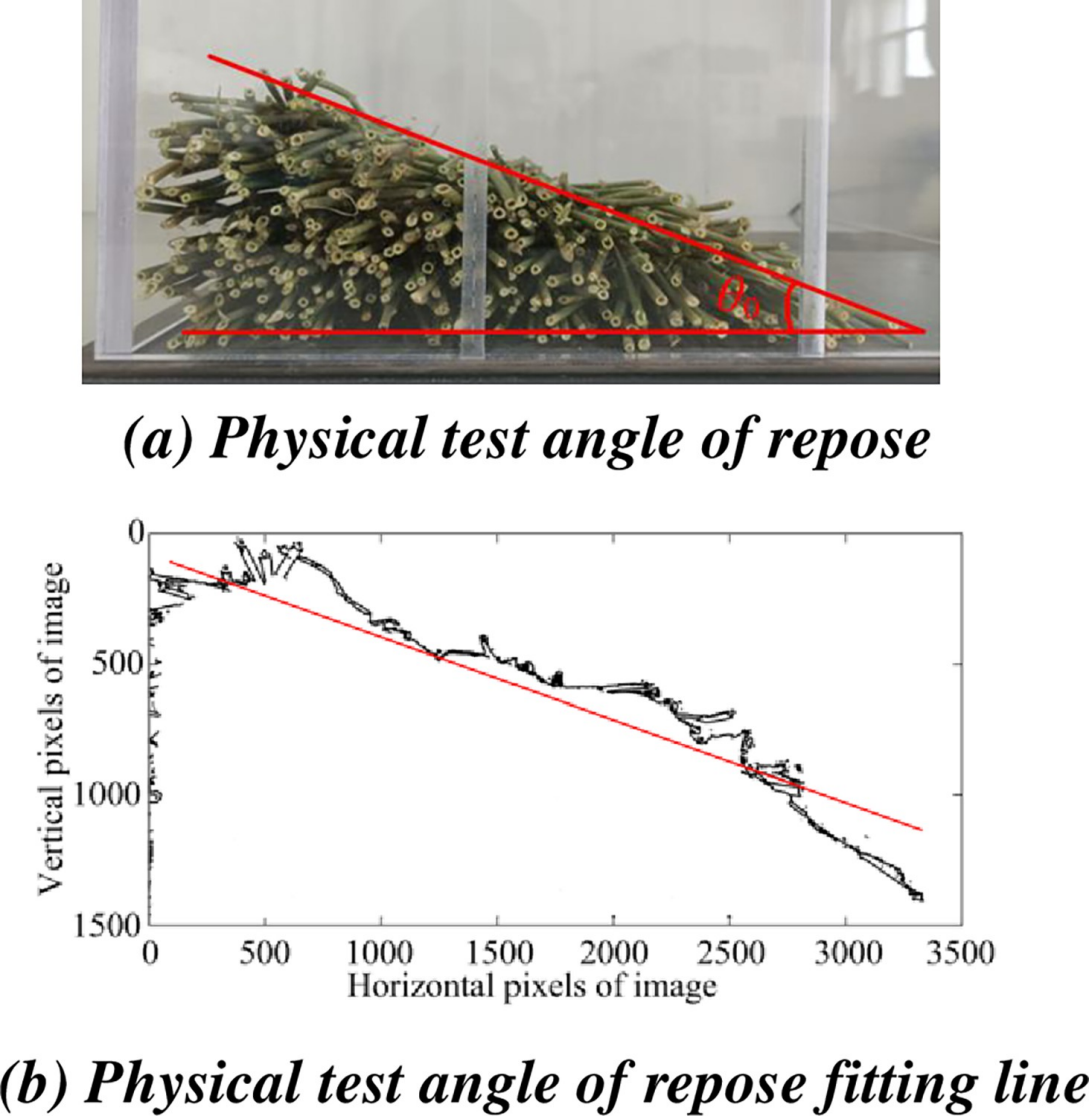
Fitting process of angle of repose of physical test. (a) Physical test angle of repose. (b) Physical test angle of repose fitting line.

*Repose angle test*. The repose angle of alfalfa stalks was measured by side wall collapse method. Acrylic plates were used to construct the device’s four walls and centre baffle, making it easy to see how the internal stalks were flowing, the bottom was made of steel plate. Under the influence of outside forces, the center baffle could freely move in the vertical direction. The test device is shown in Fig 7. 300 alfalfa stalks with a length of 200 mm were placed between the side wall and the baffle of the measuring device. The central baffle was pull off slowly while the stalks were perfectly stationary. Alfalfa stalks flowed to the side that was not blocked. After the test is completed, in order to reduce the error of manual measurement, the HD camera is selected to collect the image information of the angle of repose, and the Matlab software is used to process the HD angle of repose image. Firstly, the image information is read and processed with gray scale, then the gray scale image is binarized with appropriate threshold value and the image is filled with imfill function. After processing, there will be a lot of noise in the image. In order to extract the ideal edge contour curve, appropriate filling radius is selected for corrosion or expansion processing. Finally, the contour curve of the boundary was extracted and the image information was imported into Origin, where the pixels of the edge curve of the angle of repose were extracted manually, the coordinate values of the horizontal and vertical direction of the axis were set, and the two-dimensional map was drawn and linear fitting was carried out. Finally, the slope of the fitted line was converted into the angle of the angle of repose of the alfalfa stem. The fitting process of angle of repose for physical test is shown in Fig 8.

The test was carried out for five times, and discovering that the alfalfa stalks’ angle of repose is 33.45°, with a coefficient of variation of 0.98%.

**Fig 9:**
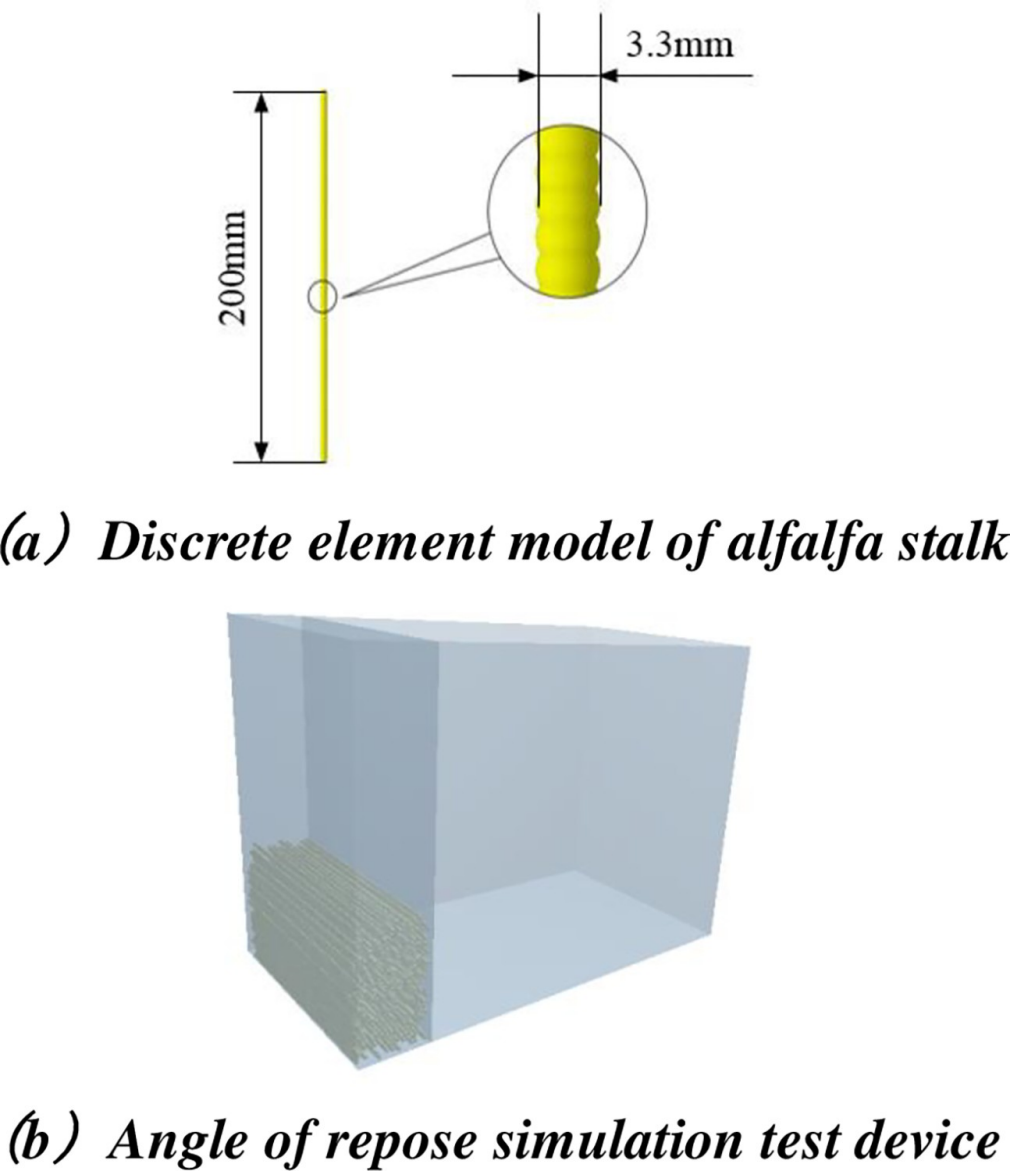
Simulation model of alfalfa stalk repose angle. (a) Discrete element model of alfalfa stalk. (b) Angle of repose simulation test device.

*Establishment of EDEM repose angle simulation model*. The Hertz-Mindlin (no slip) model is used in the EDEM software to carry out the simulation test for the angle of repose. The alfalfa stalk is similar to a cylinder. 100 spherical particles with a diameter of 3.3 mm are sequentially arranged to create a 200 mm long alfalfa stalk model by the method of spherical particle combination method. A measuring device with the same structural size as the physical test is established. 300 stalks are generated statically. After the stalks are stacked stably, the moving baffle moves upward at the speed of 0.05mm/s, and the stalks collapse to form the repose angle. The established model of the alfalfa stalk, the angle of repose simulation device, and the angle of repose formed θ_1_ are shown in Fig 9.

**Fig 10:**
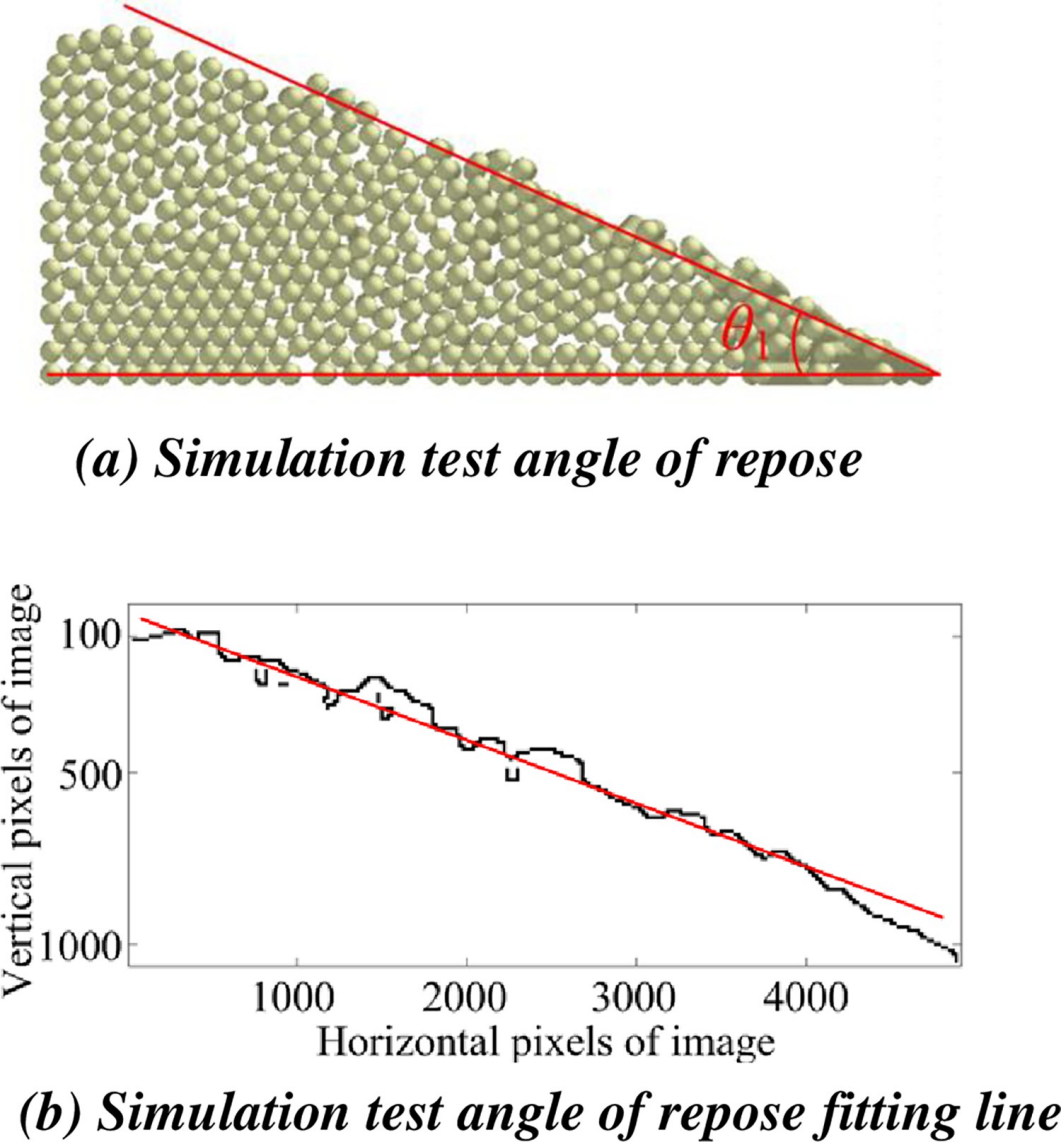
Fitting process of angle of repose of simulation test. (a) Simulation test angle of repose. (b) Simulation test angle of repose fitting line.

The fitting process of the simulation angle of repose is shown in Fig 10.

#### Design of test method

*Plackett-Burman design*. A N = 11 Plackett-Burman design table is used, with five dummy variables reserved for error analysis to screen the contact factors that have a significant effect on the angle of repose. The high and low levels (+1 and -1) of the six contact parameters are determined according to the measured values of the physical test, and carried out the angle of repose simulation test.

*The steepest ascent test*. The steepest ascent test was used for the significant parameters that have been obtained to determine the region close to the optimal value. The non-significant basic contact parameters are taken as the intermediate level in the Placket-Burman test, and the significant parameters are gradually increased according to the set step size. The repose angle simulation analysis was carried out, the change of the repose angle obtained from the simulation test and the relative error of the physical test are recorded, and the optimal value adjacent area was determined according to the change trend of the relative error.

*Box-Behnken design*. Based on the results of Plackett-Burman test, the steepest ascent test, and the design principle of Box-Behnken Design (BBD), the high level value, the center point and the low level value of the proximity region of the optimal value of the significance parameter are taken as the high, middle and low levels (+1, 0 and -1) of BBD test design. The angle of repose simulation test was carried out. The non-significant parameters in the simulation model were the same as those in the steepest ascent test.

## Results and discussion

### Results of the Plackett-Burman test

Test parameters of Plackett-Burman are shown in Table 1, the test scheme and results are shown in Table 2, and the significance test results are shown in Table 3.

**Table 1:** Plackett-Burman test value range.

Parameters	Level	
	Low (-1)	High (1)
Restitution coefficient of alfalfa-alfalfa *x*_1_	0.35	0.55
Static friction coefficient of alfalfa-alfalfa *x*_2_	0.4	0.6
Rolling friction coefficient of alfalfa-alfalfa *x*_3_	0.05	0.25
Restitution coefficient of alfalfa-steel *x*_4_	0.3	0.5
Static friction coefficient of alfalfa-steel *x*_5_	0.3	0.7
Rolling friction coefficient of alfalfa-steel *x*_6_	0.1	0.3

**Table 2:** Plackett-Burman test scheme and results.

Serial number	*x*_1_	*x*_2_	*x*_3_	*x*_4_	*x*_5_	*x*_6_	Repose angle *θ*_1_ (°)
1	0.55	0.6	0.05	0.5	0.7	0.3	32.43
2	0.35	0.6	0.25	0.3	0.7	0.3	31.92
3	0.55	0.4	0.25	0.5	0.3	0.3	33.87
4	0.35	0.6	0.05	0.5	0.7	0.1	36.49
5	0.35	0.4	0.25	0.3	0.7	0.3	30.36
6	0.35	0.4	0.05	0.5	0.3	0.3	35.48
7	0.55	0.4	0.05	0.3	0.7	0.1	31.32
8	0.55	0.6	0.05	0.3	0.3	0.3	25.26
9	0.55	0.6	0.25	0.3	0.3	0.1	28.69
10	0.35	0.6	0.25	0.5	0.3	0.1	35.35
11	0.55	0.4	0.25	0.5	0.7	0.1	37.42
12	0.35	0.4	0.05	0.3	0.3	0.1	31.51

**Table 3:** Significance test of Plackett-Burman test results.

Source	Sum of Squares	df	Mean Square	F-value	P-value
Model	126.60	6	21.10	15.92	0.0040
*x*_1_	12.24	1	12.24	9.23	0.0288*
*x*_2_	8.04	1	8.04	6.06	0.0571
*x*_3_	2.18	1	2.18	1.65	0.2555
*x*_4_	85.23	1	85.23	64.28	0.0005**
*x*_5_	7.97	1	7.97	6.01	0.0578
*x*_6_	10.94	1	10.94	8.25	0.0349*
Residual	6.63	5	1.33		

**Fig 11:**
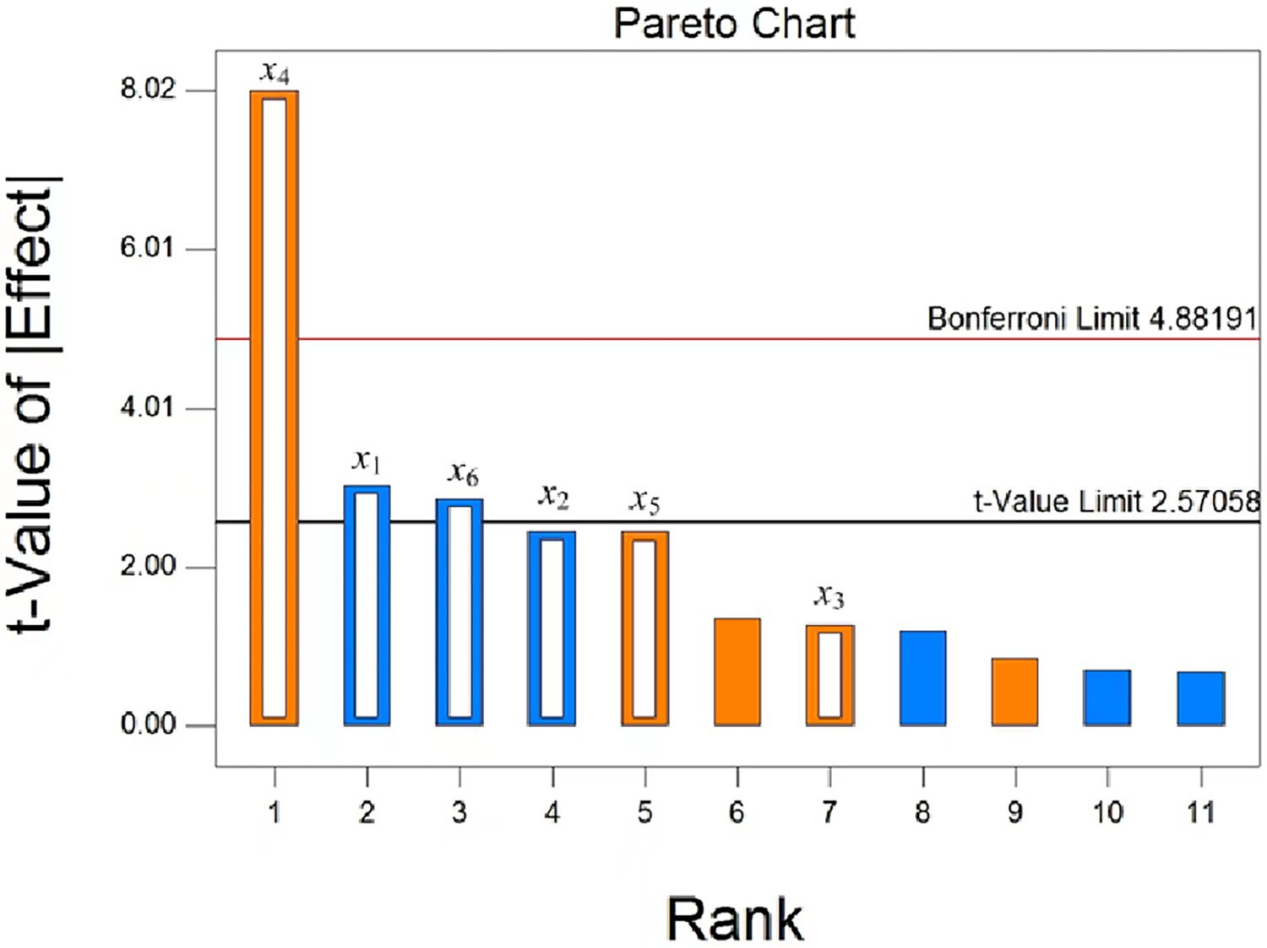
Pareto chart of Plackett-Burman experiment.

It can be known from Table 3 and Fig 11, the primary and secondary order of various factors affect the repose angle θ_1_ was x_4_, x_1_, x_6_, x_5_, x_2_ and x_3_. Because the P values of x_4_, x_1_ and x_6_ were less than 0.05, indicating that the factors had significant effects on the target. The Pareto Chart’s t-value test provided the same result, with the effect value of x_4_ on the target being positive and the effect values of x_1_ and x_6_ being negative.

### Results of the steepest ascent test

The steepest ascent test scheme and results are shown in Table 4.

**Table 4:** Steepest ascent test scheme and results.

No.	*x*_1_	*x*_4_	*x*_6_	Repose angle *θ*_1_/ (°)	Relative error *δ*/ (%)
1	0.35	0.3	0.1	27.33	18.3
2	0.4	0.35	0.15	31.21	6.7
3	0.45	0.4	0.2	34.76	3.92
4	0.5	0.45	0.25	36.73	9.81
5	0.55	0.5	0.3	38.94	16.41

It could be seen from the steepest ascent test results that the relative error was the smallest at level 3, so the optimal range was near level 3. Therefore, level 3 was taken as the center point, and level 2 and level 4 were taken as the low level and high level respectively for the Box-Behnken test of contact parameters.

### Results of Box-Behnken test

In the Design-Expert 12.0 software, the three-factor, three-level Box-Behnken test Design is carried out with Steepest ascent test level 3 as the level 0, level 2 and level 4 as the low level and high level, respectively, and five sets of repeats are set at the central level. A total of 17 groups of alfalfa stem repose Angle simulation tests were required, and the other non-significant parameters were consistent with the Steepest ascent test. The horizontal coding of the physical parameters of significance is shown in Table 5. The test scheme and results are shown in Table 6.

**Table 5:** Significant parameter level coding table.

Level	*x*_1_	*x*_4_	*x*_6_
-1	0.4	0.35	0.15
0	0.45	0.4	0.2
+1	0.5	0.45	0.25

**Table 6:** Box Behnken test scheme and results.

Serial number	*x*_1_	*x*_4_	*x*_6_	Repose angle *θ*_1_ (°)	Error *δ*/ (%)
1	0.4	0.4	0.15	30.67	8.31
2	0.4	0.4	0.25	32.81	1.91
3	0.5	0.4	0.15	32.32	3.38
4	0.5	0.4	0.25	34.44	2.96
5	0.45	0.35	0.15	30.52	8.76
6	0.45	0.35	0.25	31.38	6.19
7	0.45	0.45	0.15	32.67	2.33
8	0.45	0.45	0.25	34.23	2.33
9	0.4	0.35	0.2	28.81	13.87
10	0.5	0.35	0.2	29.55	11.66
11	0.4	0.45	0.2	30.71	8.20
12	0.5	0.45	0.2	35.33	5.62
13	0.45	0.4	0.2	32.78	2.00
14	0.45	0.4	0.2	33.02	1.29
15	0.45	0.4	0.2	32.64	2.42
16	0.45	0.4	0.2	32.42	3.08
17	0.45	0.4	0.2	32.79	1.97

The Design-Expert 12.0 software was used to perform variance analysis on the results of Box-Behnken test. The analysis results are shown in Table 7.

**Table 7:** Variance analysis of Box-Behnken test results.

Source	Sum of Squares	df	Mean Square	F-value	P-value
Model	45.65	9	5.07	17.27	0.0005**
*x*_1_	9.33	1	9.33	31.77	0.0008**
*x*_4_	20.10	1	20.10	68.42	<0.0001**
*x*_6_	5.58	1	5.58	18.99	0.0033**
*x*_1_ *x*_4_	3.76	1	3.76	12.81	0.0090**
*x*_1_ *x*_6_	1.0E-004	1	1.0E-004	3.4E-004	0.9858
*x*_4_ *x*_6_	0.12	1	0.12	0.42	0.5930
$x_{1}^{2}$	1.70	1	1.70	5.78	0.0472*
$x_{4}^{2}$	4.17	1	4.17	14.19	0.0070**
$x_{6}^{2}$	0.91	1	0.91	3.10	0.1217
Residual	2.06	7	0.29		
Lack of fit	1.86	3	0.62	12.77	0.1620
Pure error	0.19	4	0.05		
Cor total	47.71	16			

**Fig 12:**
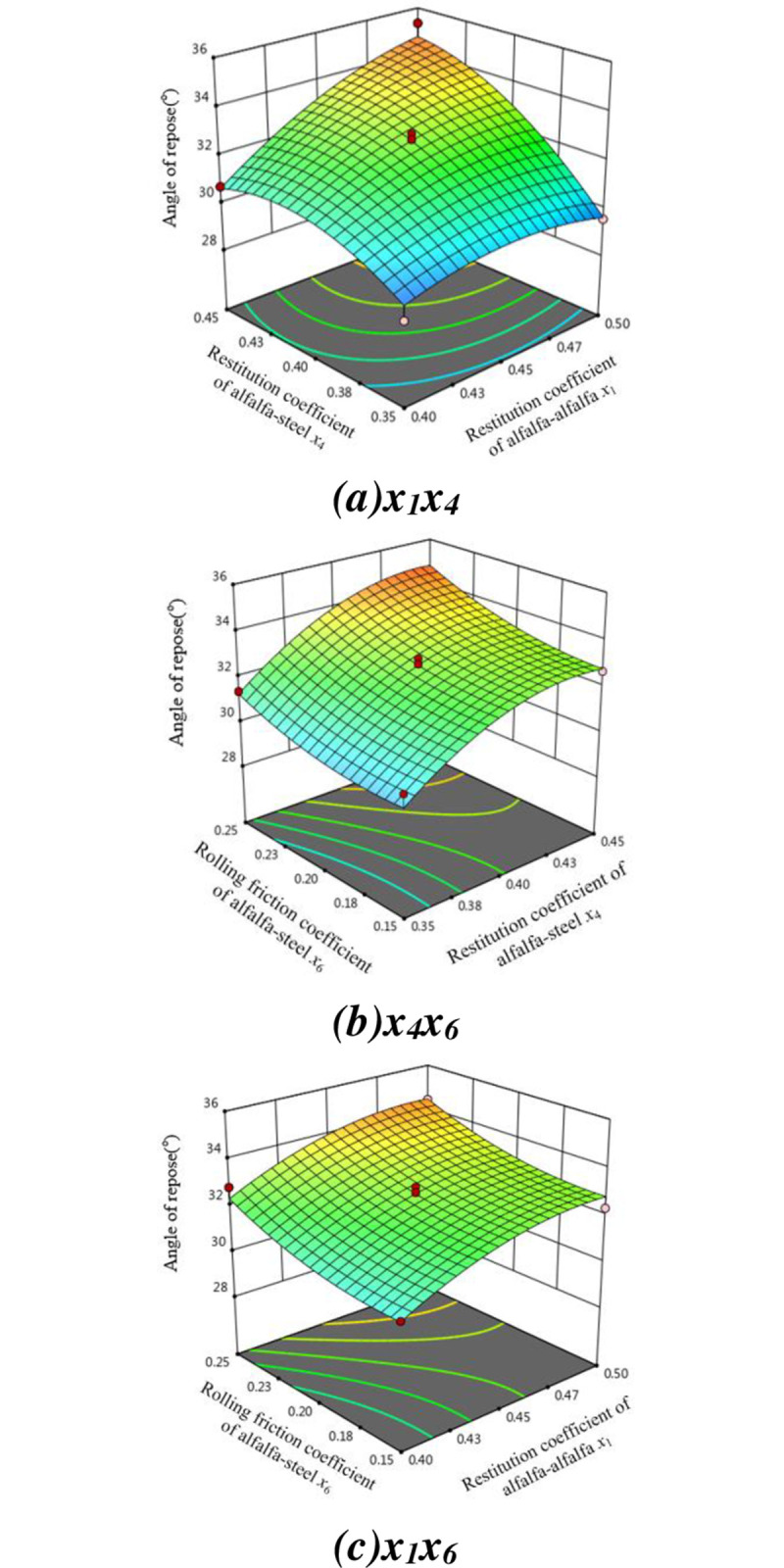
Response surface of alfalfa stalk repose angle. (a) x_1_x_4_. (b) x_4_x_6_. (c) x_1_x_6_.

The response surface of the interaction between the factors to the angle of repose is shown in Fig 12.

It can be known from the analysis results that x_1_, x_4_, x_6_, x_1_x_4_, $x_{4}^{2}$ have very significant effects on the angle of repose, $x_{1}^{2}$ has significant effect on the angle of repose, other terms have no significant impact on the angle of repose. This angle of repose fitting regression model (P = 0.0005) is significant, and the mismatch term (P = 0.16) is not significant, indicating that the model is well fitted and no mismatch occurs. The determination coefficient R_2_ = 0.9569, the correction determination coefficient R_adj_ = 0.9015, which is very close to 1, and the coefficient of variation C.V. = 1.68% is low, indicating that this model can accurately reflect the real situation and can be used to predict the angle of repose. The second order regression model of the simulated repose angle of alfalfa stalk and three significant parameters is obtained, and the equation is:

$$\begin{array}{l} \theta_{1}=-25.43+95.4x_{1}+161.5x_{4}-84.8x_{6}+388x_{1}x_{4}\\ -2x_{1}x_{6}+70x_{4}x_{6}-254x_{1}^{2}-398x_{4}^{2}+186x_{6}^{2} \end{array}$$ (13)

### Parameter optimization

The optimization module in Design-Expert 12.0 software is used to optimize the regression model 13. The physical test value of the angle of repose (33.45°) is defined as the optimization target value, and x_1_, x_4_ and x_6_ are the optimization objects. According to the Placket-Burman and steepest ascent tests, the ranges of x_1_, x_4_ and x_6_ have been determined to be 0.4~0.5, 0.35~0.45 and 0.15~0.25 respectively. Therefore, the objective function and constraint function of the optimization problem are as follows:

$$\left\{ \begin{array}{l} \theta_{1}(x_{1},x_{4},x_{6})=33.45\\ \left\{ \begin{array}{l} 0.4\leq x_{1}\leq 0.5\\ 0.35\leq x_{4}\leq 0.45\\ 0.15\leq x_{6}\leq 0.25 \end{array}\right. \end{array}\right.$$ (14)

The optimal parameter combinations of alfalfa stalk-alfalfa stalk restitution coefficient x_1_, alfalfa stalk-steel restitution coefficient x_4_ and alfalfa stalk-steel rolling friction coefficient x_6_ are 0.46, 0.42 and 0.2, respectively. The calculated fitting value of the repose angle is 33.47°, and the relative error with the average value of the repose angle in the physical test is 0.06%, indicating that the established regression model for predicting the repose angle of alfalfa stalk is accurate.

### Verification test

In order to verify the accuracy and feasibility of the calibrated contact parameters, the optimal parameter combination is selected for the significant parameters of the repose angle, and the non-significant parameters are taken as the middle value of the physical test measurement range. The angle of repose simulation test is carried out, and three repeated tests are carried out. The test parameter values are shown in Table 8.

**Table 8:** Contact parameters of simulation verification test for the repose angle of alfalfa stalk discrete element at primary florescence.

Parameters	Value
Poisson’s ratio of alfalfa stalk	0.38
Poisson’s ratio of steel	0.30
Shear modulus of alfalfa stalk/MPa	15.8
Shear modulus of steel/Pa	7.94×10^10^
Density of alfalfa stalk/(kg·m^-3^)	1045
Density of steel/(kg·m^-3^)	7850
Restitution coefficient of alfalfa-alfalfa	0.46
Static friction coefficient of alfalfa-alfalfa	0.5
Rolling friction coefficient of alfalfa-alfalfa	0.15
Restitution coefficient of alfalfa-steel	0.42
Static friction coefficient of alfalfa-steel	0.5
Rolling friction coefficient of alfalfa-steel	0.2

The average value of the simulated angle of repose obtained is 33.61°, and the relative error between the average value of the simulated angle of repose and physical test is 0.48%. The angle of repose obtained by simulation test and physical test are very close, indicating that the calibrated physical parameters of the alfalfa stalk are accurate and feasible.

## Conclusion

In this study, the variation range of static friction coefficient, rolling friction coefficient and restitution coefficient of alfalfa stalk at the primary florescence was obtained by using the inclined plane method, high-speed camera technology and other physical test methods. The angle of repose is determined by the repose angle test. The discrete element method and response surface method were used to calibrate the contact parameters of alfalfa stalks, and the angle of repose was used as the response value. The contact parameters of alfalfa stalks were analyzed, screened and optimized by Placket-Burman, steepest ascent and Box-Behnken tests. The following conclusions are obtained:

1. The analysis of variance of Plackett-Burman test show that restitution coefficient of alfalfa stalk-alfalfa stalk, the restitution coefficient of alfalfa stalk-steel and rolling friction coefficient of alfalfa stalk-steel have significant effects on the angle of repose, while the other parameters have no significant effects on the angle of repose.
2. The results of the steepest ascent test and Box-Behnken test show that the regression model of the repose angle has good precision and reliability. The optimal combination of alfalfa stalk-alfalfa stalk, alfalfa stalk-steel restitution coefficient and rolling friction coefficient of alfalfa stalk-steel is 0.46, 0.42 and 0.2.
3. The verification experiment shows that the relative error between the simulation result and the measured value is 0.48%. That means the calibrated contact parameters are accurate and reliable, and the established discrete element model of alfalfa stalk at the primary florescence is similar to the physical characteristics of the actual stalk, which can provide a reference for the design and discrete element simulation of forage conveying mechanism.

In this study, the parameters of the discrete element model of alfalfa stalk in the early flowering period were determined by the Angle of repose test, which provided a method for the establishment of discrete element simulation model of stalk crops. Due to the complexity of the biological materials of alfalfa stem and the limitation of simulation methods, the sampling of the angle of repose test was only represented by the materials of the middle section of alfalfa stem at the initial flowering stage with the length of 100mm and the diameter of 3mm. The parameters determined have a certain applicable range. Systematic studies will be conducted on the stems at different growth stages and different parts in the future.

## Supporting information

S1 Data (DOC)
