# Supplementary material for: Calibration and test of contact parameters for alfalfa stalk at primary florescence based on discrete element method
Source: PLoS One. 2024 Aug 29;19(8):e0303064. doi: 10.1371/journal.pone.0303064 (PMC11361690; doi:10.1371/journal.pone.0303064)
Supplement: S1 Data — (DOC) [file pone.0303064.s001.doc]

**Materials and methods**

| Data in the article | Raw data |
| --- | --- |
| Average diameter of alfalfa stem root was 3.48mm | 3.41,3.62,3.54,3.58,3.52,3.36,3.39,3.55,3.43,3.39 |
| Stem length of alfalfa was 134.6mm | 133.9,135.1,134.2,133.8,135.2,134.9,135.1,136.3,133.7,134.2 |
| Median diameter of alfalfa stem was 3.16mm | 3.21,3.11,3.27,3.11,3.12,3.09,3.23,3.28,3.08,3.11 |
| Middle length of alfalfa stem was 156.1mm | 155.8,157.3,154.2,157.1,154.6,158.3,153.9,157.4,155.5,157.2 |
| Average diameter of alfalfa stem neck was 2.92mm | 2.87,2.76,3.12,3.01,2.96,3.05,2.91,2.88,2.74,2.93 |
| Alfalfa stem neck length was 163.3mm | 162.7,163.7,163.5,161.2,165.8,162.9,161.4,164.9,165.7,162.1 |

**Determination of material parameters**

| Data in the article | Raw data |
| --- | --- |
| Static friction coefficient between stem and stem of alfalfa was 0.4~0.6 | 0.412,0.513,0.596 |
| Static friction coefficient of alfalfa stem and steel plate was 0.3~0.7 | 0.324,0.517,0.686 |
| Alfalfa stem - Rolling friction coefficient of alfalfa stem was 0.05~0.25 | 0.052,0.186,0.247 |
| Rolling friction coefficient of alfalfa stem and steel plate was 0.1~0.3 | 0.11,0.22,0.29 |
| Collision recovery coefficient of alfalfa stem - alfalfa stem was 0.35~0.55 | 0.348,0.425,0.547 |
| Collision recovery coefficient of alfalfa stem and steel plate was 0.3~0.5 | 0.304,0.427,0.546 |

**Poisson's ratio and shear modulus**

| Data in the article | Raw data |
| --- | --- |
| Poisson's ratio 0.5 | 0.47,0.52,0.54,0.48,0.49 |
| Modulus of elasticity49.6MPa | 50.2,47.7,51.3,52.5,46.9,47.4,50.2,50.3,49.1,50.3 |
| Shear modulus18.3MPa | 17.1,17.2,20.3,18.2,18.3,18.5,17.1,20.2,17.8,18.3 |

**Repose angle model**

| Data in the article | Raw data |
| --- | --- |
| The average repose angle of alfalfa stalk physical test was 33.45^o^ | 33.45,33.49,33.47,33.43,33.41 |

**Verification test**

| Data in the article | Raw data |
| --- | --- |
| Average repose angle of simulation test was 33.61^o^ | 32.86,33.69,34.28 |

***The rest of the data was obtained through only one test, so the data in the article is the raw data.***
